# Assessing asthma symptoms in children: qualitative research supporting the development of the *Pediatric Asthma Diary—Child (PAD-C)* and *Pediatric Asthma Diary—Observer (PAD-O)*

**DOI:** 10.1186/s41687-023-00639-y

**Published:** 2023-10-20

**Authors:** Helena Bradley, Claire Trennery, Amy M. Jones, Aoife Lydon, Frances White, Rebecca Williams-Hall, Rob Arbuckle, Erin Tomaszewski, Vivian H. Shih, John Haughney, Amanda Eisen, Tonya Winders, Stephen Joel Coons, Sonya Eremenco, the Patient-Reported Outcome Consortium’s Pediatric Asthma Working Group, Linda Nelsen, Maggie Tabberer, Maria Mattera, Asha Lehane, Lucy Morgan, Jerry Krishnan

**Affiliations:** 1 Adelphi Values Ltd, Bollington, Cheshire UK; 2 GlaxoSmithKline, London, UK; 3 AstraZeneca, Gaithersburg, MD USA; 4 NHS Greater Glasgow and Clyde R&I, Queen Elizabeth University Hospital, Glasgow, UK; 5 Allergy and Asthma Network, Vienna, VA USA; 6 Global Allergy and Airways Patient Platform, Vienna, Austria; 7 Patient-Reported Outcome Consortium, Critical Path Institute, Tucson, AZ USA

**Keywords:** Pediatric asthma, Patient-reported outcome (PRO), Observer-reported outcome (ObsRO), Qualitative, Development, Diary, Interview, Measurement, Symptoms, Signs

## Abstract

**Background:**

Pediatric asthma has been identified by regulators, clinicians, clinical trial sponsors, and caregivers as an area in need of novel fit-for-purpose clinical outcome assessments (COAs) developed in accordance with the U.S. Food and Drug Administration’s (FDA’s) regulatory guidance for evaluating clinical benefit in treatment trials. To address this gap, the Patient-Reported Outcome (PRO) Consortium’s Pediatric Asthma Working Group has continued development of 2 COAs to assess asthma signs and symptoms in pediatric asthma clinical trials to support efficacy endpoints: a PRO measure, the *Pediatric Asthma Diary—Child* (*PAD-C*) for children 8–11 years old (y.o.) and an observer-reported outcome measure, the *Pediatric Asthma Diary-Observer* (*PAD—O)* for caregivers of children 4–11 y.o. This qualitative research aimed to generate evidence regarding the content validity of the *PAD-C* and *PAD-O*.

**Methods:**

Semi-structured combined concept elicitation and cognitive interviews were conducted with a diverse sample of U.S. participants (15 children 8–11 y.o. and 30 caregivers of children 4–11 y.o.). All children had clinician-diagnosed mild to severe asthma. Interviews explored the experience of pediatric asthma and assessed the understanding and relevance of both measures. Interviews were conducted across 3 iterative rounds to allow for modifications.

**Results:**

Concept elicitation findings demonstrated that the core sign/symptom and impact concepts assessed in the *PAD-C* (cough, hard to breathe, out of breath, wheezing, chest tightness, and nighttime awakenings/symptoms) and *PAD-O* (cough, difficulty breathing, short of breath, wheezing, and nighttime awakenings/signs) correspond to those most frequently reported by participants; concept saturation was achieved. All *PAD-C* and *PAD-O* instructions and core items were well understood and considered relevant by most participants. Feedback from participants, the Pediatric Asthma Working Group, advisory panel, and FDA supported modifications to the measures, including addition of 1 new item to both measures and removal of 1 caregiver item.

**Conclusions:**

Findings provide strong support for the content validity of both measures. The cross-sectional measurement properties of both measures and their user experience and feasibility in electronic format will be assessed in a future quantitative pilot study with qualitative exit interviews, intended to support the reliability, construct validity, final content, and, ultimately, FDA qualification of the measures.

**Supplementary Information:**

The online version contains supplementary material available at 10.1186/s41687-023-00639-y.

## Abstract (plain language summary)

Pediatric asthma is one of the most common chronic diseases in children. However, there are problems of underdiagnosis, poor disease management, and undertreatment for many pediatric asthma patients, pressuring healthcare systems worldwide. Evaluating asthma symptoms is an important part of the development of treatments for pediatric asthma. However, there are few clinical outcome assessments (COAs) developed in line with regulatory guidance to directly assess symptom severity and evaluate the benefit of new treatments in children with asthma. In this study, we continued the development of the *Pediatric Asthma Diary—Child (PAD-C)* and the *Pediatric Asthma Diary—Observer (PAD-O)*, according to regulatory guidance, to assess asthma signs and symptoms in children 4 through 11 years old and address this unmet need. The study aimed to explore the experience of pediatric asthma and assess how well-understood and relevant the measures are. Three rounds of qualitative interviews were conducted with 15 children 8 through 11 years old and 30 caregivers of children 4 through 11 years old with asthma. Results show that both measures are well-understood and assess the relevant and important aspects of pediatric asthma reported by children and caregivers. Findings provide evidence supporting the *PAD-C* and *PAD-O* as measures of symptom severity and their future use in pediatric asthma treatment trials. Further research is underway to evaluate their measurement properties and assess the user experience and feasibility of electronic completion, to ultimately support the *PAD-C* and *PAD-O* in an ongoing COA qualification process by the United States Food and Drug Administration.

**Supplementary Information:**

The online version contains supplementary material available at 10.1186/s41687-023-00639-y.

## Background

As a chronic inflammatory disease of the airways, pediatric asthma is characterized by recurrent episodes of shortness of breath, wheeze, chest tightness, and cough. These episodes are typically associated with expiratory airflow limitation that may resolve spontaneously or in response to medication [1]. Pediatric asthma is recognized as the most common chronic disease in children [2, 3]; however, prevalence is increasing globally and issues of underdiagnosis, poor disease management, and undertreatment continue to persist [4]. As a result, pediatric asthma remains a critical area of unmet need and poses a substantial global burden on healthcare systems [5].

International guidelines issued by the Global Initiative for Asthma (GINA) state that the long-term goals of asthma management are to achieve good symptom control and to minimize future risk of exacerbations, persistent airflow limitation, and side effects of treatment [1]. The achievement of good symptom control necessitates the assessment of asthma symptoms; however, there are poor correlations between objective measures of asthma severity typically used in clinical trials (e.g., forced expiratory volume in 1 s and peak expiratory flow) and patients’ self-reported experience [6–8]. The assessment of asthma symptoms is a critical component in the development of treatments for pediatric asthma and to ease the burden on children and their families. Therefore, to ensure the patient perspective of asthma is accurately represented and assessed in clinical research, there is a need for novel clinical outcome assessments (COAs) to directly assess symptom severity and evaluate clinical benefit in pediatric asthma populations [9, 10].

Symptoms of asthma are most appropriately assessed using patient-reported outcome (PRO) measures, since only persons with asthma can feel and self-report on many symptoms. However, as young children (i.e., ≤ 7 years old [y.o.]) may not be able to reliably self-report symptom experience, pediatric asthma trials can involve the collection of PRO data from older children (i.e., ≥ 8 y.o.) on asthma symptoms and observer-reported outcome (ObsRO) data from parents/caregivers on observable asthma-related signs for younger children [9]. Although recent efforts to develop COAs in pediatric asthma exist [11], there is still a lack of fit-for-purpose COAs developed in accordance with United States (U.S.) Food and Drug Administration’s (FDA’s) evidentiary expectations for evaluating clinical benefit in pediatric asthma clinical trials [12]. During previous interactions between FDA and the PRO Consortium’s Asthma Working Group during qualification of the Asthma Daytime Symptom Diary (ADSD) and the Asthma Nighttime Symptom Diary (ANSD) for adolescent and adult populations [13, 14], FDA feedback noted the measurement gap in pediatric populations and requested the development of novel COAs to assess asthma symptoms in a broader range of asthma patients (i.e., < 12 y.o.) in clinical studies.

To address this, Merck Sharpe & Dohme Corporation, a member of Critical Path Institute’s (C-Path’s) PRO Consortium [15], contributed draft versions of 2 COAs for use in pediatric asthma clinical trials to assess the signs and symptoms of mild to severe persistent asthma: a PRO measure designed for completion by children 8–11 y.o. (originally named the Child Asthma Diary [CAD]); and an ObsRO measure designed for completion by parents/caregivers of children 4–11 y.o. (originally named the Observer Asthma Diary [OAD]) [16]. Initial development of the measures was informed by multiple stages of qualitative research, including a targeted literature review, input by expert scientific advisors, 3 phases of concept elicitation interviews, and 2 phases of cognitive interviews with the respective target populations. However, initial FDA feedback to Merck raised concerns regarding adequacy of the evidence for the content validity of the CAD and OAD in the planned context of use. As a result, the PRO Consortium’s Pediatric Asthma Working Group embarked on further development of the CAD and OAD, with the intention of submitting for COA qualification by FDA for the assessment of asthma sign and symptom severity in children with asthma (i.e., < 12 y.o.) in pediatric asthma clinical trials [17]. A reanalysis of Merck’s original qualitative data collected as part of the initial development of the draft CAD and OAD was conducted to address FDA’s feedback. Based on this reanalysis of the original data, the draft CAD and OAD were subsequently modified and renamed the *Pediatric Asthma Diary—Child* (*PAD-C)* and *Pediatric Asthma Diary—Observer* (*PAD-O)*, respectively. FDA accepted the *PAD-C* and *PAD-O* into the Drug Development Tool (DDT) COA Qualification Program on June 13, 2017. FDA input has therefore been sought at key points throughout the development and qualification process [17] and has been outlined throughout this article where applicable.

The *PAD-C* and *PAD-O* are intended to be used to derive co-primary or secondary endpoints in pediatric asthma clinical trials to establish clinical benefit and support product-specific labeling claims. This article summarizes the qualitative research conducted to continue the development of the *PAD-C* and *PAD-O* and to generate qualitative evidence supporting their content validity in accordance with FDA regulatory guidance [10, 18, 19].

## Methods

### Study design

**Fig. 1:**
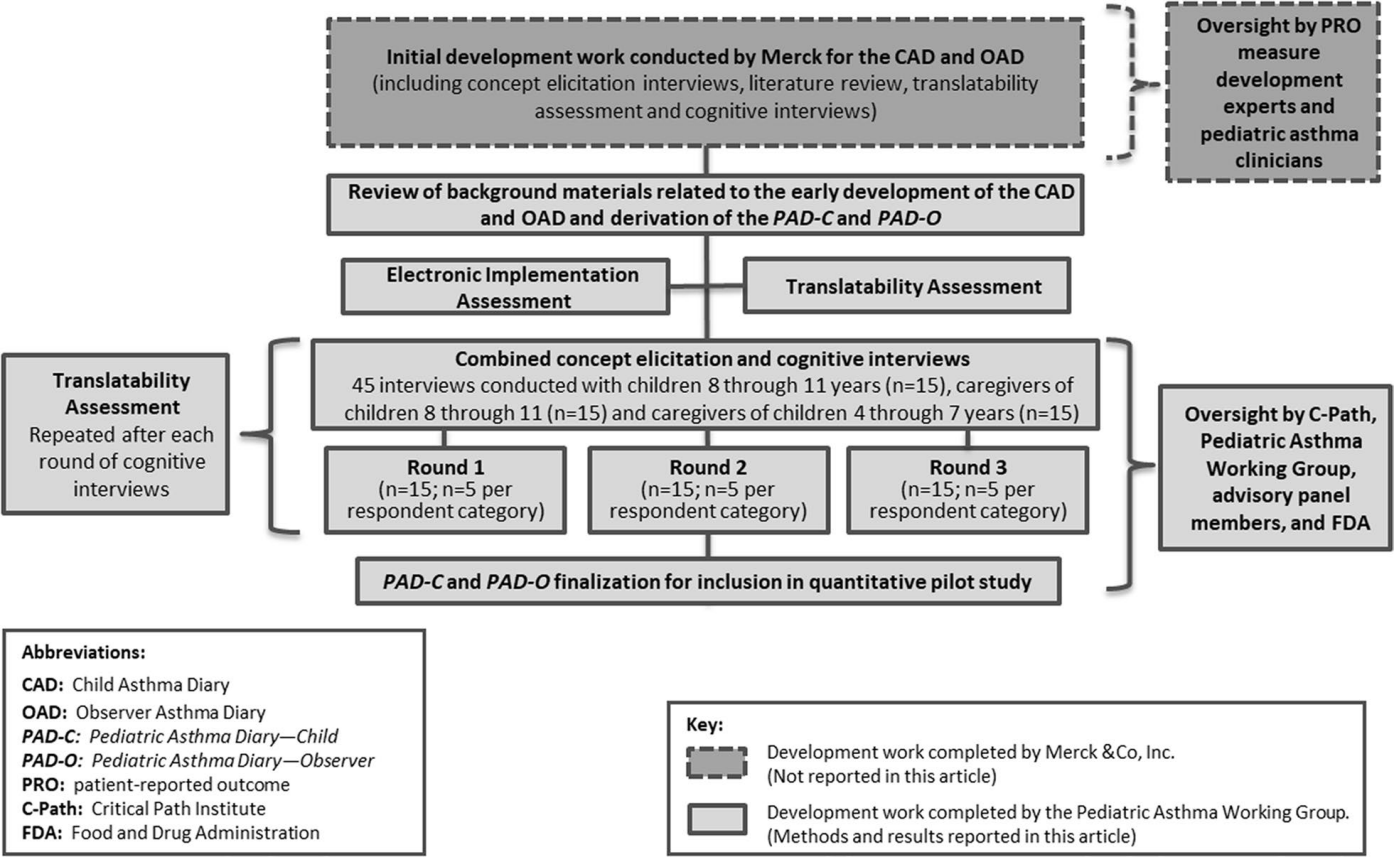
Overview of study design

Figure 1 provides an overview of the qualitative research conducted to support the development of the *PAD-C* and *PAD-O*.

At key points throughout the process, input was obtained from the Pediatric Asthma Working Group, C-Path scientists, the advisory panel (J.K, J.H, A.E, T.W), and FDA’s Qualification Review Team. A translatability assessment was conducted on the measures following each round of interviews to ensure that any modifications to the wording used would be suitable for future translation into other languages.

### Initial draft *PAD-C* and *PAD-O*

A number of changes occurred to the CAD and OAD to create the modified *PAD-C* and *PAD-O*. Prior to the study reported here, the PRO Consortium’s Pediatric Asthma Working Group and Adelphi Values made additional refinements to the *PAD-C* and *PAD-O* ahead of inclusion and testing in the qualitative interviews. Changes included rearranging and streamlining the *PAD-C* and *PAD-O* training guides and simplifying the terminology used in the instructions and item wording of each measure. This section describes the initial draft *PAD-C* and *PAD-O* tested in the Round 1 concept elicitation and cognitive interviews.

The *PAD-C* and *PAD-O* are designed to be completed twice daily and include a Morning Diary (completed once daily upon waking up to start the day) to assess nighttime awakenings and nighttime asthma symptom severity and a Bedtime Diary / Evening Diary (completed once daily before going to bed) to assess daytime symptom severity. Both measures include a Training Guide that all participants must read prior to completing the *PAD-C* or *PAD-O* to aid understanding of the diaries.

The draft *PAD-C* (7-item Morning Diary and 12-item Bedtime Diary) has been developed for use in children with asthma 8–11 y.o. to assess self-reported asthma symptom severity. The draft *PAD-O* (9-item Morning Diary and 12-item Evening Diary) has been developed for use in caregivers of children with asthma 4–11 y.o. to assess caregiver-reported asthma sign/symptom severity. When completing the *PAD-O*, the caregiver can also consider input from other informants (e.g., the child, siblings, teachers, babysitters, and spouses/partners) regarding observable asthma signs and symptoms.

The *PAD-C* Bedtime Diary assesses the severity of 5 core asthma symptoms (cough, hard to breathe, out of breath, wheezing, and chest tightness) and the *PAD-O* Evening Diary assesses the severity of 4 core observable asthma signs and symptoms (cough, difficulty breathing, shortness of breath, and wheezing). Note that chest tightness was not included within the *PAD-O* as it was found to not be an observable concept that can be reliably reported by caregivers in the previous qualitative research [16]. Due to difficulty with feasibility of children or caregivers reporting on the severity of individual symptoms during the night, a global assessment of nighttime asthma symptom or sign severity is included within the *PAD-C* Morning Diary and *PAD-O* Morning Diary, respectively. The morning diaries also assess presence of nighttime awakenings, which is considered a clinically relevant marker for asthma control and symptom severity [1]. The asthma sign and symptom concepts included within the *PAD-C* and *PAD-O* are assessed in terms of presence (nighttime awakenings), intensity (cough, nighttime asthma symptom severity), or frequency (cough, difficulty breathing, shortness of breath, wheezing, and chest tightness [*PAD-C* only]), to provide an assessment of sign and symptom severity which is widely recognized as needed to demonstrate clinical benefit in pediatric asthma treatment trials.

Additional items included in the *PAD-C* and *PAD-O* to assess other asthma-relevant measurement concepts are: difficulty falling asleep (Morning Diary), activity limitations (Bedtime Diary / Evening Diary), and rescue medication use for both rescue inhalers and nebulizers (Morning Diary and Bedtime Diary / Evening Diary). Single items designed to assess global daytime asthma symptom severity are included in the Bedtime Diary / Evening Diary only, to support analyses during measure development. In the *PAD-O*, items are included to capture the sources of information used by caregivers when responding to items for informational purposes only, in addition to 1 item added at FDA’s request assessing whether caregivers check on their child during the night (Morning Diary only).

Items in the *PAD-C* are answered using a 4- or 5-level verbal rating scales (VRS) with text descriptors for each response option paired with colored boxes of increased shading, or via “Yes/No” response options. Items in the *PAD-O* are answered using a 5- or 6-level VRS, or via “Yes/No/I don’t know” response options. Number entry fields are also used for the rescue inhaler and nebulizer items in both measures.

### Concept elicitation and cognitive interviews

Combined semi-structured concept elicitation and cognitive interviews were conducted across 3 iterative rounds to evaluate modifications made to the *PAD-C* and *PAD-O*.

#### Recruitment

Forty-five participants were targeted for inclusion in the interviews, including 15 children 8–11 y.o., 15 parents/caregivers of children 4–7 y.o., and 15 parents/caregivers of children 8–11 y.o. These subgroups allowed for development and testing of the *PAD-C* and *PAD-O* in narrower age groupings to help account for developmental differences in children [9, 20]. Participants were recruited from 5 different U.S. locations (Chicago, IL; Baltimore, MD; New Orleans, LA; Pittsburgh, PA; St. Louis, MO) with the assistance of a third-party recruitment agency via referral by general practitioners, pediatricians, and respiratory specialists. Child participants were required to be 8–11 y.o., have a clinician-confirmed diagnosis of asthma as defined by national or international asthma guidelines (i.e., GINA [1], National Asthma Education and Prevention Program [NAEPP] [21]) for at least 1 year, have received/filled a prescription for asthma medication in the last year, and have experienced symptoms of asthma in the 3 weeks prior to screening. Caregivers were required to be at least 18 years of age and a parent/caregiver of a child 4–11 y.o. with a clinician-confirmed diagnosis of pediatric asthma, who had received/filled a prescription for asthma medication in the last year and had experienced signs or symptoms of asthma in the 3 weeks prior to screening.

Participants were excluded if they (or their child if a caregiver) had a diagnosis of a condition other than asthma (not including allergies or rhinitis) that affected lung function (e.g., bronchiectasis, chronic sinusitis, cystic fibrosis) or any other significant condition that would impact ability to take part in the study.

Recruitment quotas for the following characteristics were used to ensure a sociodemographically and clinically diverse sample reflective of respondents typically enrolled in pediatric asthma clinical trials: age, sex, ethnicity, race, time since diagnosis, asthma control (i.e., well-controlled and not well-controlled [22]), exacerbations, and medication use.

#### Interview procedure

The research was conducted in accordance with the Declaration of Helsinki and ethical approval and oversight were provided by Copernicus Group Independent Review Board (CGIRB), an independent ethical review board in the U.S. (IRB number: 20200606). All data were handled in accordance with Health Insurance Portability and Accountability Act (HIPAA) guidelines and the European General Data Protection Regulation (GDPR) for the security and privacy of health data.

All participants provided written informed consent (or parental permission and participant assent in the case of participants 8–11 y.o.) before their participation in the study. Semi-structured interviews lasting approximately 60 min were conducted by trained qualitative researchers via Microsoft Teams or by telephone. All interviews were audio recorded and transcribed verbatim.

In each round, interviews included an introduction (5 min), concept elicitation (5 min), and cognitive interview (50 min) sections. A brief concept elicitation component was included at the start of the interview, to explore the experience of pediatric asthma and evaluate whether the *PAD-C* and *PAD-O* adequately assess the core symptoms reported by participants. Since comprehensive concept elicitation work was completed by Merck during initial development activities, this section was deliberately brief to allocate more time to the cognitive evaluation of the *PAD-C* and *PAD-O*. Following concept elicitation, participants were asked to complete a paper version of the *PAD-C* (children) or *PAD-O* (caregivers) using a “think aloud” method to vocalize their thoughts as they read each instruction and completed each item. In-depth cognitive interview questions were then used to explore the relevance and understanding of the diary items, instructions, response scales, and recall periods.

#### Analysis

A qualitative analysis plan was developed a priori to define the coding process, subgroup analyses, and presentation of results. All interview data were analyzed using qualitative analysis methods and ATLAS.ti software [23].

Concept saturation, defined as the point at which no new relevant or important information emerges with the collection of more data [10], was evaluated to ensure that the concepts elicited by participants during the concept elicitation portion of the interview had been fully explored. Saturation analyses were conducted for the child and caregiver samples separately by dividing participants into 3 equal groups according to the chronological order in which they were interviewed. Saturation was said to be achieved if no new concepts emerged within the final group of interviews (i.e., Round 3 interviews).

## Results

### Sample characteristics

A total of 45 participants were included across 3 rounds of interviews. Fifteen interviews were conducted with children 8–11 y.o. and 30 interviews were conducted with caregivers of children 4–11 y.o. All children had clinician-diagnosed mild to severe asthma.

Table 1 summarizes the sociodemographic and clinical characteristics of children participating or being represented by a caregiver in the qualitative interviews. Sociodemographic characteristics of caregivers are presented in Additional file 1: Table 1. Overall, the majority of pre-specified recruitment quotas were met or only narrowly missed, and there was good representation of characteristics in both child and caregiver samples for each age group.

**Table 1:** Child sociodemographic and clinical characteristics (n = 30)

Characteristic	Child being represented by caregiver		Child participating in study
	4–7 y.o. (n = 15)	8–11 y.o (n = 3)^1^	8–11 y.o (n = 15)
*Age (years)*			
Mean	4.8	10	9.3
Min, Max	4, 7	8, 11	8, 11
*Sex, n (%)*^2^			
Male	10 (66.7%)	2 (66.7%)	9 (60.0%)
Female	5 (33.3%)	1 (33.3%)	6 (40.0%)
*Ethnicity, n (%)*			
Non-Hispanic or Latino	10 (66.7%)	**–**	11 (73.3%)
Hispanic or Latino (of any race)	5 (33.3%)	3 (100%)	4 (26.7%)
*Race, n (%)*			
Black/African American	4 (26.7%)	**–**	5 (33.3%)
White	3 (20.0%)	**–**	4 (26.7%)
Multi-racial	4 (26.7%)	1 (33.3%)	3 (20.0%)
Asian	1 (6.7%)	**–**	1 (6.7%)
Other: reported Hispanic as race	3 (20.0%)	2 (66.67%)	2 (13.3%)
*Asthma control according to participant score on C-ACT, n (%)*^3^			
Well-controlled (C-ACT score: ≥ 20)	8 (53.3%)	–	7 (46.7%)
Not well-controlled (C-ACT score: ≤ 19)	7 (46.7%)	3 (100%)	8 (53.3%)
*Participant experience of an exacerbation in the past two weeks, n (%)*			
No, did not experience an exacerbation	9 (60.0%)	1 (33.3%)	8 (53.3%)
Yes, experienced a moderate exacerbation	4 (26.7%)	2 (66.7%)	4 (26.7%)
Yes, experienced a severe exacerbation	2 (13.3%)	1 (33.3%)^4^	3 (20.0%)
*Type of treatment currently receiving for management of Asthma, n (%)*^5^			
Step 2	6 (40.0%)	**–**	7 (40.0%)
Step 3	5 (33.3%)	1 (33.3%)	3 (20.0%)
Step 4	4 (26.7.%)	2 (66.7%)	5 (33.3%)

^1^n = 3 children 8–11 years old (y.o.) were represented by caregivers who participated in an interview, but the children were not interviewed themselves

^2^All participants’ identified gender was the same as their sex

^3^C-ACT = Childhood Asthma Control Test

^4^One participant experienced both moderate and severe exacerbations

^5^Step-wise categories of medication use are based on GINA guidelines [1]

### Concept elicitation results

The symptoms most frequently reported by children during the concept elicitation section of the interviews correspond to the 5 core symptom concepts assessed in the *PAD-C* Bedtime Diary; cough, difficulty breathing, and chest tightness were reported by all child participants (n = 15/15, 100%), and shortness of breath and wheezing were reported by almost all (n = 14/15, 93.3%; see Table 2). These symptoms were elicited in each round of interviews and equally across both levels of asthma control (well-controlled and not well-controlled).

**Table 2:** Summary of asthma signs/symptoms and impacts reported during concept elicitation in Round 1, 2, and 3 interviews (N = 45)

Concept	Participant type	Example participant quote
*Asthma sign/symptoms*		
Cough (N = 45/45, 100%)	Child 8–11 y.o. (n = 15/15, 100%) Caregiver of child 4–11 y.o. (n = 30/30, 100%)	06-20-M-8-WC-P: *“…I would really like start coughing, like coughing real bad…I will really cough a lot…and I'll like need my…asthma pump…”* 11-30-F-34-WC-CG: *“She will just cough all day when it's real bad. Um, there have been times at night where she will cough and keep her up, uh, cough to the point of throwing up.”*
Difficulty breathing (N = 45/45, 100%)	Child 8–11 y.o. (n = 15/15, 100%) Caregiver of child 4–11 y.o. (n = 30/30, 100%)	27-40-F-11-NWC-P: *“Asthma, it's hard to breathe. It's like when you can't do much things 'cause it's hard to breathe…”* 09-20-F-35-NWC-CG: *“Well typically she'll always, um, grab her chest or around her throat area and say that it's difficult to breathe or she's having a hard time breathing…”*
Wheezing (n = 44/45, 97.8%)	Child 8–11 y.o. (n = 14/15, 93.3%) Caregiver of child 4–11 y.o. (n = 30/30, 100%)	18-50-M-9-NWC-P: *“I hear a loud wheezing noise.”* 19-40-F-31-WC-CG: *“…So he usually has like wheezing and short of breath, um, especially like certain times of the night sometimes. And also like if he's, um, at school and he's doing too much activity or something, he can experience symptoms like that.”*
Shortness of breath (n = 42/45, 93.3%)	Child 8–11 y.o. (n = 14/15, 93.3%) Caregiver of child 4–11 y.o. (n = 28/30, 9.33%)	23-40-M-9-NWC-P: *“Uh, I get out of breath quickly.”* 08-40-F-38-NWC-CG: *“…He has a hard time kind of just catching his breath, um, just seems out of breath.”*
Chest tightness (n = 19/45, 42.2%)	Child 8–11 y.o. (n = 15/15, 100%) Caregiver of child 4–11 y.o. (n = 4/30, 13.3%)	09-20-F-10-NWC-P*: “My chest tighten up.”* 15-40-F-40-WC-CG*: “…Um, he feels from what I can see and what he can describe is like a tightness of the chest. So kind of like the, the walls of his body kind of closing in.”*
*Domains of impacts on daily life*		
Physical activity (N = 45/45, 100%)	Child 8–11 y.o. (n = 15/15, 100%) Caregiver of child 4–11 y.o. (n = 30/30, 100%)	10-40-F-9-WC-P: *“…you can't, you can't like play like a normal person. Like you can't like play without having to stop to take your pump or something.”* 23-40-F-36-NWC-CG: *“If just—if he’s not feeling well, then there’s just—he can’t go play or run.”*
Sleep (n = 44/45, 97.8%)	Child 8–11 y.o. (n = 15/15, 100%) Caregiver of child 4–11 y.o. (n = 29/30, 96.7%)	27-40-F-11-NWC-P: *“Well like sometimes I wake up from my sleep like more than once a week. Like I just wake up 'cause it's like, like I can't breathe when I'm sleeping. So I wake up.”* 09-20-F-35-NWC-CG: *“Um, yes. There has been times that it has been very difficult for her to go to sleep, um, especially when she's having a really, really bad flare-up…”*
Social functioning (n = 18/45, 40.0%)	Child 8–11 y.o. (n = 7/15, 46.7%) Caregiver of child 4–11 y.o. (n = 11/30, 36.7%)	03-40-M-8-WC-P: *“Uh, the worst thing is that like if like it's—um, so like in the summertime, if I play with my friends and my family and we usually run around if we're having like a water balloon fight or water gun fight. That I have to take a break and I can't play anymore until like it goes away and starts to slow down.”* 23-40-F-36-NWC-CG: *“…Um, I mean there's times that he can't do things that he likes to do as a kid, you know, in school trying to play with his friends. And when he's not feeling well, he just doesn’t.”*
Emotional wellbeing (n = 17/45, 37.8%)	Child 8–11 y.o. (n = 5/15, 33.3%) Caregiver of child 4–11 y.o. (n = 12/30, 40.0%)	13-10-M-11-NWC-P: *“When I do wheeze, it makes like—to me it makes a loud noise, like again a whistle noise. And it will—sometimes it will scare me because when, uh, I do wheeze that means I again have trouble breathing.”* 20-50-F-24-NWC-CG: *“Um, where he is short of breath, where he's to the point where he's crying because he's—I guess it's like an anxiety feeling for him where he can't breathe.”*
School (n = 4/45, 8.9%)	Child 8–11 y.o. (n = 3/15, 20.0%) Caregiver of child 4–11 y.o. (n = 1/30, 3.3%)	14-10-M-8-NWC-P: *“About my breathing well and not going to school because I can't go to school because I'm sick of the asthma.”* Interviewer: *“…how do these symptoms affect your child?”* 23-40-F-36-NWC-CG: *“Um, with his school work. You know, trying to focus sometimes.”*

Participant IDs are presented as follows: participant number, site number, sex (M = Male; F = Female), participant age (y.o. = years old), level of asthma control (WC = Well-controlled; NWC = Not Well-controlled), and participant type (P = pediatric participant; CG = caregiver participant)

Similarly, the signs and symptoms most frequently reported by caregivers during the concept elicitation section of the interviews correspond to the 4 observable signs and symptoms assessed in the *PAD-O* Evening Diary; cough, difficulty breathing, and wheezing were reported by all caregivers (n = 30/30, 100%), and shortness of breath was reported by most (n = 28/30, 93.3%; see Table 2). Children and caregivers also reported other asthma symptoms including general congestion (n = 3 caregivers), tiredness (n = 3 caregivers), flushed face (n = 2 caregivers), and nasal congestion (n = 2 children).

Children and caregivers discussed how asthma impacted their/their child’s daily life. Impacts on physical activity (n = 15/15 children, 100%; n = 30/30 caregivers, 100%) and sleep (n = 15/15 children, 100%; n = 29/30 caregivers, 96.7%) were reported most frequently by children and caregivers, both of which are assessed by items in the *PAD-C* and *PAD-O*. Impacts on social functioning (n = 7/15 children, 46.7%; n = 11/30 caregivers, 36.7%), emotional wellbeing (n = 5/15 children, 33.3%; n = 12/30 caregivers, 40.0%), and school (n = 3/15 children, 20.0%; n = 1/30 caregiver, 3.3%; see Table 2) were also reported by children and caregivers. In terms of asthma treatments, all children (n = 15/15, 100%) and all but 1 caregiver (n = 29/30, 96.7%) reported the use of a rescue inhaler. Nebulizer use (n = 8/15 children, 53.3%; n = 20/30 caregivers, 66.7%) and maintenance inhaler use (n = 5/15 children, 33.3%; n = 18/30 caregivers, 60.0%) were also reported.

Concept saturation was achieved after the first 2 rounds of child interviews, by which point the majority of signs/symptoms and impact domains had been elicited. This included the core symptom concepts assessed by the *PAD-C* (cough, difficulty breathing, shortness of breath, wheezing, and chest tightness) and *PAD-O* (cough, difficulty breathing, shortness of breath, and wheezing), as well as impacts on physical activity and sleep (Additional file 1: Tables 2 and 3, respectively). Note that, nighttime awakenings were spontaneously reported for the first time in the final round of child interviews; however, nighttime awakenings were reported by 13 additional children when probed across the 3 rounds, supporting relevance of the concept to this patient population.

### Cognitive interview results for the *PAD-C* and *PAD-O*

For the cognitive interviews, the Pediatric Asthma Working Group and Adelphi Values divided each of the measures into core, supplementary, and developmental items. “Core items” assess the severity of key signs, symptoms, and impacts of pediatric asthma intended for inclusion in scoring of the measure. “Supplementary items” assess other optional asthma-relevant concepts intended to supplement the measures when used in clinical trials, and “developmental items” are intended for testing purposes during development of the measures. A distinct sample of 15 participants took part in each round of cognitive interviews; 5 children completed the *PAD-C* and 10 caregivers completed the *PAD-O* in each round. Across all 3 rounds of cognitive interviews, *PAD-C* and *PAD-O* instructions and items were generally well understood and considered relevant. See Additional file 1: Figs. 1, 2, 3, 4, 5, 6, 7 and 8—for an overview of understanding and relevance across the 3 rounds of child and caregiver interviews.

The 3 iterative rounds of cognitive interviews supported refinement of the *PAD-C* and *PAD-O,* with revisions to the instructions and items implemented after each round, as summarized in Table 3 (*PAD-C*) and Table 4 (*PAD-O*). Modifications made were based on feedback from participants, the Pediatric Asthma Working Group, C-Path scientists, the advisory panel, FDA scientists, and the translatability assessments. Updates were generally applied across both measures where applicable, with the aim of promoting consistency and comprehensiveness.

**Table 3:** Summary of key modifications made to the *PAD-C* following each round of interviews

	Key modifications made following Round 1 child interviews	Key modifications made following Round 2 child interviews	Key modifications made following Round 3 child interviews
Training guide	Training guide sections were removed to reduce respondent burden	A new section was added to the Training Guide to reinforce that children should give the diary back to their parent/caregiver after completing the items, as this instruction was often missed by children within the diaries	Wording in the Training Guide was updated to reflect the possibility that diary answers could be shared with the doctor
Instructions	An instruction in the Morning Diary and Bedtime Diary explaining when to answer the questions was removed to reduce respondent burden and avoid repetition of the same information included in the Training Guide	There were no key modifications made to the *PAD-C* instructions following Round 2 child interviews	An instruction in the Bedtime Diary was updated to specify “bedtime” as the end of “today” more clearly
Core items	Item stems were updated for items assessing frequency of cough and wheeze to ensure phrasing consistency across the frequency items Response options for the global nighttime asthma symptom severity item were updated to allow respondents to indicate that no symptoms were experienced to support future potential symptom-free days endpoints	The response scale for the item assessing cough intensity was updated to an alternative response scale tested in Round 2 interviews, following feedback from participants that they could have a frequent cough that is not severe The definition of “wheezing” in the Training Guide was simplified and added to the item assessing wheezing to facilitate participant understanding A definition of asthma symptoms included in the Training Guide was added to the global nighttime asthma symptom severity item as a separate sentence to facilitate participant understanding One of the response options for the global nighttime asthma symptom severity item was updated to specify asthma symptoms, to reinforce the context of asthma and facilitate understanding	There were no key modifications made to the *PAD-C* core items following Round 3 child interviews
Supplementary items	Rescue inhaler items were updated to include a definition of rescue inhaler, in addition to child-friendly terms to improve participant understanding New supplementary items assessing whether the child has a nebulizer were added to both diaries, as not all participants in Round 1 interviews reported having a nebulizer to use for asthma treatment. Similar to other existing items, instructions for skip logic were included and tested to ensure that when completed on an electronic device, those who do not have a nebulizer (i.e., those who select “No”) can skip the following nebulizer items to reduce respondent burden Wording in the nebulizer items was updated to facilitate understanding; an alternative term for nebulizer was added To reinforce the recall period and ensure accurate data collection, relevant recall periods were added to items assessing amount of rescue inhaler used and number of times nebulizer used. The response option and item wording were also updated to clarify that participants should report the total number of puffs or times used	A definition of rescue inhaler in the rescue inhaler items was refined and shortened and an alternative term for “rescue” was removed due to lack of understanding The nebulizer items were updated to ensure that they collect data on rescue medication use only as some participants in Round 2 interviews stated that nebulizers were used for both rescue and maintenance medication	Due to lack of understanding in Round 3 interviews, a definition of rescue inhaler in the rescue inhaler items was updated to include wording that was previously tested and well understood in Round 2 interviews Wording included in the nebulizer items specifying rescue medication use was simplified to similar wording that was tested and well understood in Round 2 interviews, to facilitate participant understanding
Developmental items	Response options for the global daytime asthma symptom severity item were updated to allow respondents to indicate that no symptoms were experienced to support assessment of change	A definition of asthma symptoms included in the Training Guide was added to the global daytime asthma symptom severity item as a separate sentence to facilitate participant understanding One of the response options for the global daytime asthma symptom severity item was updated to specify asthma symptoms, to reinforce the context of asthma and facilitate understanding	There were no key modifications made to the child developmental items following Round 3 child interviews

**Table 4:** Summary of key modifications made to the *PAD-O* following each round of interviews

	Key modifications made following Round 1 caregiver interviews	Key modifications made following Round 2 caregiver interviews	Key modifications made following Round 3 caregiver interviews
Training Guide	A section in the Training Guide outlining how to answer the diary was updated to include the additional example of “family friends” as people that caregivers can receive input from regarding their child’s asthma symptoms	Relevant Training Guide sections were updated to allow for both single and multiple-observer completion, providing greater flexibility and inclusivity of a broader range of family circumstances in future clinical trials. Multiple-observer completion allows for a maximum of 2 observers to share completion of the *PAD-O*, if single observer completion is not feasible	As for the *PAD-C* Training Guide, wording in the *PAD-O* Training Guide was updated to reflect the possibility that diary answers could be shared with the doctor
Instructions	There were no key modifications made to the *PAD-O* instructions following Round 1 caregiver interviews	The Morning Diary and Evening Diary instructions were updated to allow for the possibility of both single and multiple-observer completion, to align with the updates to the Training Guide	As for the instructions in the *PAD-C* Bedtime Diary, instructions in the *PAD-O* Evening Diary were updated to specify “bedtime” as the end of “today” more clearly
Core items	There were no key modifications made to the *PAD-O* core items following Round 1 caregiver interviews	The item assessing cough intensity was updated to an alternative item and response scale tested in Round 2 interviews including the terminology “severe” based on better participant understanding As for the *PAD-C* core items, one of the response options for the global nighttime asthma symptom severity item was updated to specify asthma symptoms	There were no key modifications made to the *PAD-O* core items following Round 3 caregiver interviews
Supplementary items	As for the child supplementary items, new caregiver supplementary items assessing whether the child has a nebulizer were added to both diaries and instructions for skip logic were included and tested Wording in the caregiver nebulizer items was updated; an alternative term for nebulizer was added As for the child supplementary items, relevant recall periods were added to items assessing amount of rescue inhaler used and number of times nebulizer used. The response option and item wording were also updated to clarify that participants should report the total number of puffs or times used	As for the child supplementary items, an alternative term for “rescue” was removed from the caregiver rescue inhaler items due to lack of understanding. A definition of rescue inhaler was also added to the caregiver items As for the child supplementary items, the caregiver nebulizer items were updated to ensure that they collect data on rescue medication use only as some participants in Round 2 interviews stated that nebulizers were used for both rescue and maintenance medication	As for the child supplementary items, wording included in the caregiver nebulizer items specifying rescue medication use was simplified to similar wording that was tested and well understood in Round 2 interviews
Developmental items	The response options for the sources of information item were updated to include an additional response option for input received from family friends. Response options that were unlikely to be used during the relevant time period were removed from the Morning Dairy	As for the child developmental items, one of the response options for the caregiver global daytime asthma symptom severity item was updated to specify asthma symptoms	It was agreed to remove the caregiver developmental item from the Morning Diary asking whether caregivers check on their child during the night, as interview data showed that most caregivers regularly check on their child at night regardless of asthma symptoms The response options for the sources of information items were updated to remove an open-ended “Other” response option and to rearrange the order of the response options to reflect frequency of use by participants throughout all 3 rounds of interviews

#### Round 1

The majority of instructions and items in the *PAD-C* and the *PAD-O* were well understood, and all core items were considered relevant by most participants. Recall period instructions for the Morning Diary and Bedtime Diary / Evening Diary were understood by most children and caregivers asked. Based on interview findings, several modifications were made to the *PAD-C* and *PAD-O* training guides and overall measures, including updates to the instructions, item wording, and response options.

#### Round 2

The *PAD-C* and *PAD-O* instructions and response options were understood by most participants, and all core items were understood and relevant to the majority of participants. Almost all children and caregivers understood the recall period instructions in the Morning Diary and Bedtime Diary / Evening Diary. Despite these results, further modifications were made to the *PAD-C* and *PAD-O* training guides and overall measures, including updates to the instructions (to allow for both single and multiple-observer completion [*PAD-O* only]), item wording, and response options.

#### Round 3

No changes were suggested to the *PAD-C* and *PAD-O* core items as all items and response scales were well understood and relevant to the majority of participants. Additional sections in the *PAD-O* Training Guide relating to single and multiple-observer completion were generally well understood, and half of the caregiver sample (n = 5/10, 50.0%) indicated that they would share completion of the *PAD-O* with another caregiver (e.g., another parent or grandparent), supporting retention of multiple-observer instructions. Some further modifications were made to the *PAD-C* and *PAD-O* training guides and overall measures following Round 3 interviews, including minor updates to the recall period wording within the instructions, item wording, and response scales.

### Item finalization

**Fig. 2:**
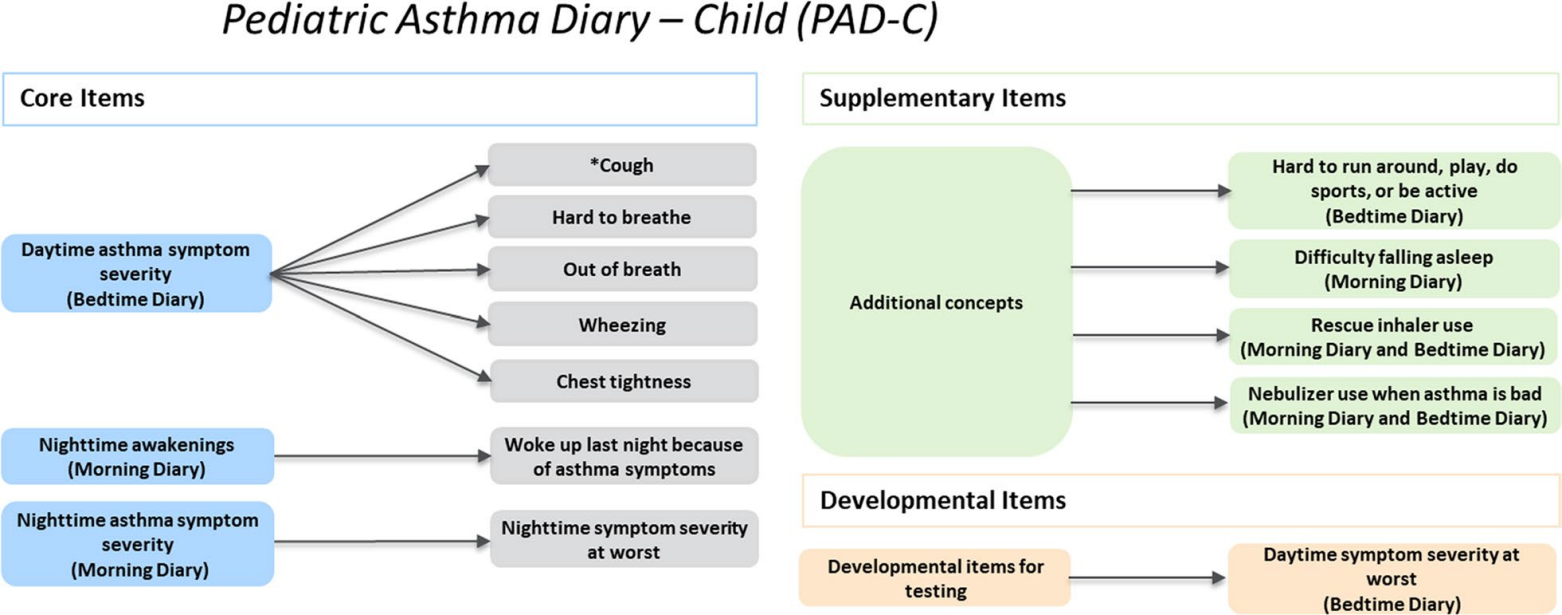
*PAD-C* draft conceptual framework. *Cough currently includes 2 items: cough frequency and cough intensity. Note: “Core Items” are intended for inclusion in the *PAD-C* scoring algorithm. “Supplementary Items” assess other optional asthma-relevant concepts intended to supplement the *PAD-C* when used in clinical trials. These items would be scored separately from the *PAD-C*. “Developmental Items” are intended for testing during *PAD-C* development.

**Fig. 3:**
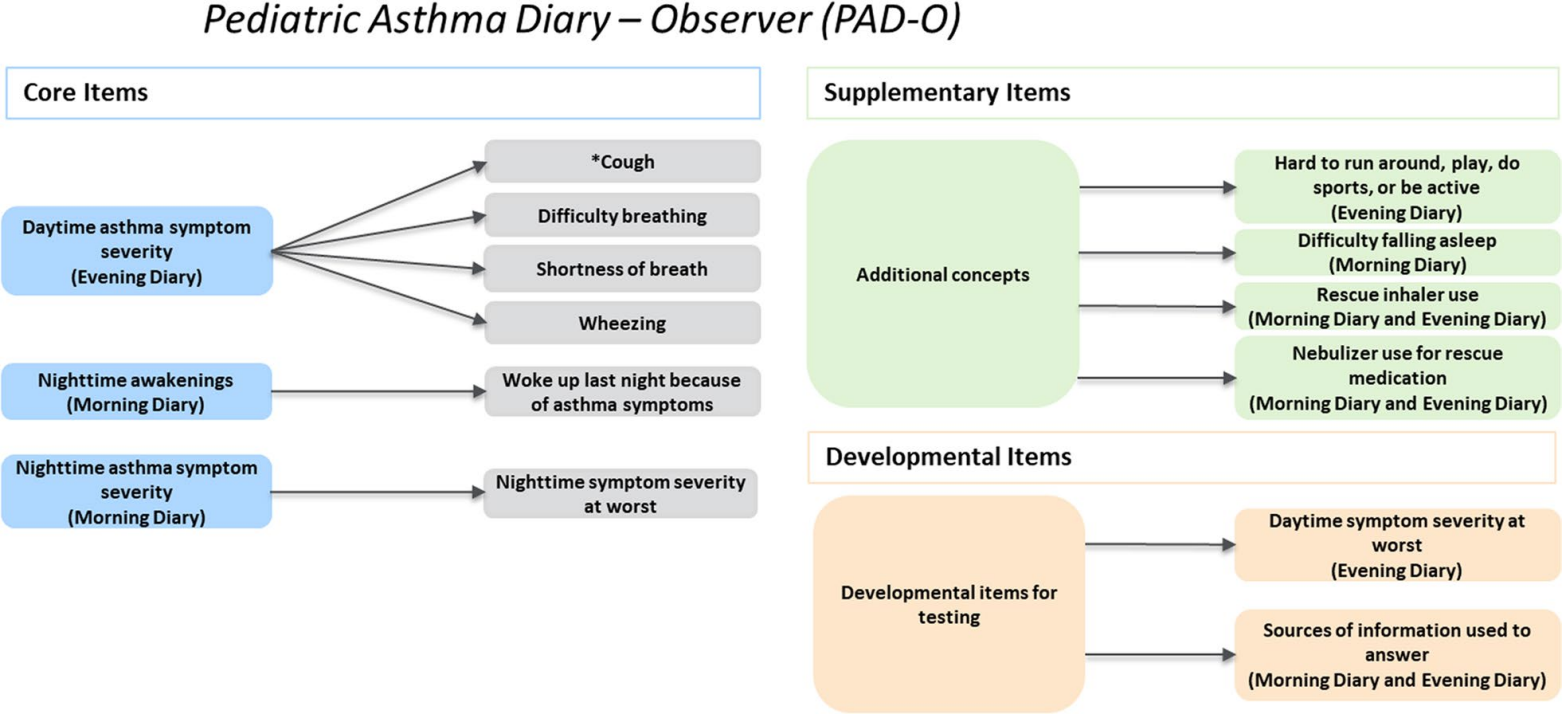
*PAD-O* draft conceptual framework. *Cough currently includes 2 items: cough frequency and cough intensity. Note: “Core Items” are intended for inclusion in the *PAD-O* scoring algorithm. “Supplementary Items” assess other optional asthma-relevant concepts intended to supplement the *PAD-O* when used in clinical trials. These items would be scored separately from the *PAD-O*. “Developmental Items” are intended for testing during *PAD-O* development

Following completion of the 3 rounds of cognitive interviews, an item finalization meeting was held with the Pediatric Asthma Working Group to discuss findings and confirm the proposed revisions to the *PAD-C* and *PAD-O*. The evidence demonstrated that both measures provided sufficient conceptual coverage of the core symptoms in pediatric asthma, and therefore it was agreed that no additional items should be added. All items tested in the Round 3 interviews were retained for both measures, except for 1 caregiver developmental item in the Morning Diary. The revisions made were reviewed and approved by the advisory panel. Following cognitive interviews, the resulting *PAD-C* consisted of 8 core items, 12 supplementary items, and 1 developmental item (Fig. 2); whereas the *PAD-O* consisted of *7* core items, 12 supplementary items, and 2 developmental items (Fig. 3).

### Conceptual frameworks

The draft conceptual frameworks for the *PAD-C* and *PAD-O* following the 3 rounds of interviews are presented in Figs. 2 and 3, respectively. These conceptual frameworks will be finalized after the completion of a planned quantitative pilot study with qualitative exit interviews.

## Discussion

Pediatric asthma has been identified by regulators and other relevant stakeholders as an area in need of novel fit-for-purpose COAs for evaluating clinical benefit in pediatric asthma treatment trials. In order to address this unmet measurement need, the *PAD-C* and *PAD-O* were accepted into FDA’s DDT COA Qualification Program [24]. The overall objective of this study was to generate qualitative evidence that the content of these measures effectively assesses the severity of the core signs and symptoms of asthma, achieved via the conduct of combined concept elicitation and cognitive interviews.

### Concept elicitation

Concept elicitation findings demonstrated that the core symptom concepts assessed in the current versions of the *PAD-C* and *PAD-O* were most frequently reported by participants, providing evidence that these measures assess the most important and relevant signs and symptoms of pediatric asthma. The most frequently reported domains of impacts on daily life were physical activity and sleep impacts (including difficulty falling asleep and nighttime awakenings), both of which are assessed by items in the *PAD-C* and *PAD-O* and are considered clinically relevant concepts directly linked to asthma symptoms. The evidence confirms that no core sign or symptom concepts were missing from the *PAD-C* or *PAD-O* and the addition of further items is not needed. The findings further substantiate existing literature highlighting the widespread and considerable impact of pediatric asthma and reinforces the need for effective treatments to achieve symptom control and accurate assessments of symptom severity [5, 13, 25].

### Cognitive interviews for the *PAD-C* and *PAD-O*

Across the 3 rounds of cognitive interviews, instructions and core items in the *PAD-C* and *PAD-O* were well understood and considered relevant by most participants, providing qualitative evidence to support their content validity. The iterative rounds of interviews strengthened the measures, with revisions to the instructions and items implemented and tested after each round. Several modifications were made following Round 1 interviews, including updates to item stems, response options, rescue inhaler and/or nebulizer item wording, and the addition of a new item to determine whether a child has a nebulizer for asthma treatment. Following Round 2 interviews, further updates were made to response options including updates to the cough intensity response scale on both the *PAD-C* and *PAD-O,* rescue inhaler and/or nebulizer item wording, and additional instructions were added to the *PAD-O* to allow for single and multiple-observer completion. Findings from Round 3 interviews supported additional changes to the rescue inhaler and/or nebulizer item wording to enhance understanding, and the item assessing whether caregivers check on their child was removed as shown in Table 4. This resulted in the current versions of the *PAD-C* and *PAD-O* at the time of publication.

Since the initiation of this research in 2016, a new electronic Pediatric Asthma Symptom Diary (ePASD) has been developed for self-completion by children 6–11 y.o., in an attempt to address the existing measurement gap in this population [11]. However, there are notable advantages of the *PAD-C* and *PAD-O* over the ePASD and other existing measures. First, the development of both a PRO measure (the *PAD-C* for completion by children 8–11 y.o.) and ObsRO measure (the *PAD-O* for completion by caregivers of children 4–11 y.o.) allows for the assessment of asthma signs and symptoms across a broader range of children with mild to severe asthma, specifically those younger than 6 y.o. Evidence from the qualitative interviews and existing literature demonstrates the importance of assessing symptom severity in children as young as 4 y.o. [1, 26], particularly as this often reflects populations included in pediatric asthma clinical trials. As such, there is a critical need for appropriate COAs with adequate evidence of being fit-for-purpose to assess asthma symptom severity in younger age groups, not purely self-reports by older children. Second, there is mixed evidence regarding the age at which a child can independently and reliably self-report, with some doubts around the appropriateness of administering PRO measures to children below the age of 8 y.o. [20, 27, 28]. The *PAD-O* was developed to avoid these potential issues in younger age groups, as caregivers are more likely to be optimal reporters of observable asthma signs and medication use for children under 8 y.o. [16]. The *PAD-O* also offers the unique ability for both single and multiple-observer completion, an addition that was supported by FDA representatives to account for a range of different caregiver and/or living situations that better reflect modern family life and allow for greater inclusivity in future pediatric asthma clinical trials. Finally, an important strength of the *PAD-C* and *PAD-O* is the pursuit of qualification as part of FDA’s DDT COA Qualification Program. Qualification ensures both measures have been developed and tested in accordance with FDA expectations and relevant guidance [10, 18, 19], including input from a diverse sample of children and caregivers from the target population with varying sociodemographic (e.g., age, sex, ethnicity, and race) and clinical characteristics (e.g., levels of asthma control, exacerbations, and medication use). This is in addition to involvement from a multidisciplinary team, COA experts, comprising representatives from 2 pharmaceutical firms, C-Path, specialist clinicians involved in the diagnosis and management of children with asthma, patient advocates, and FDA representatives.

### Study limitations

Although there was good representation of different sociodemographic and clinical characteristics in the sample, some target quotas were missed. Most notably this included children on medication Step 5 and male caregivers, although, this is likely a reflection of fewer cases of more severe asthma in children and the lack of established Step 5 treatment for children 4–5 y.o. [1], and the well-documented sex differences in research participation [29, 30] and childcare responsibilities [31, 32]. Interviews were also conducted with U.S. participants only; however, the cross-cultural suitability of the *PAD-C* and *PAD-O* was explored within the translatability assessments, and full translation and cultural adaptation of the measures for other languages will be conducted in future studies.

Additionally, interviews with children 8–11 y.o. were conducted via video call or telephone. Face-to-face interviews were initially proposed as the optimal methodology to build rapport and obtain useful insights from non-verbal cues; however, this was not feasible due to the COVID-19 pandemic and associated public health restrictions when interviews were conducted between October 2020 and July 2021. Nevertheless, research has shown comparability between face-to-face and video or telephone interviews [33, 34], and additional steps were taken to mitigate against potential issues with remote interviewing (i.e., color-coding the measures) and to promote engagement throughout the child interviews (i.e., using visual aids and creative tasks).

## Conclusion

The results from this qualitative study provide strong support for the content validity of the *PAD-C* and *PAD-O* for assessing severity of asthma signs and symptoms in children 4 through 11 y.o. with mild to severe pediatric asthma. The next steps in the development process include the migration of the measures to an electronic mode of data collection and the conduct of a quantitative pilot study with qualitative exit interviews. This continued research will aim to generate further evidence to confirm the cross-sectional measurement properties and evaluate the user experience and feasibility of electronic completion of the *PAD-C* and *PAD-O* to support progress towards their qualification in FDA’s COA Qualification Program.

### Supplementary Information

**Additional file 1**. Supplementary materials including tables and figures.

## Data Availability

The data described in this article are not publicly available in further detail beyond that provided in the manuscript and the extensive supplementary files.

## Abbreviations

*ADSD*: *Asthma Daytime Symptom Diary*
*ANSD*: *Asthma Nighttime Symptom Diary*
CAD: Child Asthma Diary
CGIRB: Copernicus Group Independent Review Board
COA: Clinical outcome assessment
C-Path: Critical Path Institute
DDT: Drug development tool
ePASD: Electronic Pediatric Asthma Symptom Diary
FDA: U.S. Food and Drug Administration
GDPR: General Data Protection Regulation
GINA: Global Initiative for Asthma
HIPAA: Health Insurance Portability and Accountability Act
HHS: Health and Human Services
NAEPP: National Asthma Education and Prevention Program
OAD: Observer Asthma Diary
ObsRO: Observer-reported outcome
*PAD-C*: *Pediatric Asthma Diary—Child*
*PAD-O*: *Pediatric Asthma Diary—Observer*
PRO: Patient-reported outcome
VRS: Verbal rating scale
U.S.: United States
y.o.: Years old
