# Supplementary material for: Assessing asthma symptoms in children: qualitative research supporting the development of the Pediatric Asthma Diary—Child (PAD-C) and Pediatric Asthma Diary—Observer (PAD-O)
Source: J Patient Rep Outcomes. 2023 Oct 20;7:104. doi: 10.1186/s41687-023-00639-y (PMC10589163; doi:10.1186/s41687-023-00639-y)
Supplement: Supplementary file 1 — Additional file 1. Supplementary materials including tables and figures. [file 41687_2023_639_MOESM1_ESM.docx]

**Supplementary Materials**

| Supplementary Table 1: Parent/caregiver participant sociodemographic characteristics (n=30) | | | | |
| --- | --- | --- | --- | --- |
| **Sample sociodemographics** | **Round 1** | **Round 2** | **Round 3** | **Total** |
|  | **Parent/Caregiver (n=10)** | **Parent/Caregiver (n=10)** | **Parent/Caregiver (n=10)** | **Parent/Caregiver (n=30)** |
| **Age (years)**  Mean  Min, Max | 38  24-51 | 34  24-45 | 42  25-70 | 37.9  24-70 |
| **^1^Sex, n (%)**  Female  Male | 7 (70.0%)  3 (30.0%) | 10 (100%)  - | 7 (70.0%)  3 (30.0%) | 24 (80.0%)  6 (20.0%) |
| **Ethnicity, n (%)**  Non-Hispanic or Latino  Hispanic or Latino (of any race) | 10 (100%)  - | 7 (70.0%)  3 (30.0%) | 5 (50.0%)  5 (50.0%) | 22 (73.3%)  8 (26.7%) |
| **Race, n (%)**  Black/African American  White  Multi-racial  Asian  Other: Reported Hispanic as race | 5 (50.0%)  4 (40.0%)  1 (10.0%)  -  - | 3 (30.0%)  1 (10.0%)  1 (10.0%)  2 (20.0%)  3 (30.0%) | 3 (30.0%)  1 (10.0%)  2 (20.0%)  1 (10.0%)  3 (30.0%) | 11 (36.7%)  6 (20.0%)  4 (13.3%)  3 (10.0%)  6 (20.0%) |
| **Highest level of education, n (%)**  College or university degree  High School Diploma or GED  Some high school  Some years of college  Grad or professional degree | 4 (40.0%)  4 (40.0%)  -  1 (10.0%)  1 (10.0%) | 7 (70.0%)  2 (70.0%)  -  1 (10.0%)  - | 2 (20.0%)  3 (30.0%)  3 (30.0%)  1 (10.0%)  1 (10.0%) | 13 (43.3%)  9 (30.0%)  3 (10.0%)  3 (10.0%)  2 (6.7%) |
| **Work status**  Working full-time  Working part-time  Unemployed  Homemaker  Retired | 6 (60.0%)  3 (30.0%)  -  1 (10.0%)  - | 7 (70.0%)  2 (20.0%)  1 (10.0%)  -  - | 5 (50.0%)  1 (10.0%)  2 (20.0%)  1 (10.0%)  1 (10.0%) | 18 (60.0%)  6 (20.0%)  3 (10.0%)  2 (6.7%)  1 (3.3%) |
| **^2^Living status, n (%)**  Living with spouse or domestic partner  Living with your children  Living with other family members  Living with parent  Other: Shared custody | 7 (70.0%)  4 (40.0%)  2 (20.0%)  -  - | 8 (80.0%)  4 (40.0%)  -  -  - | 6 (60.0%)  7 (70.0%)  -  1 (10.0%)  1 (10.0%) | 21 (70.0%)  15 (50.0%)  2 (6.7%)  1 (3.3%)  1 (3.3%) |
| **Relationship to child, n (%)**  Parent  Grandparent | 10 (100%)  - | 10 (100%)  - | 8 (80%)  2 (20%) | 28 (93.3%)  2 (6.7%) |
| **Number of nights child sleeps in the same house over a 2-week period, n (%)**  14  7  12  10 | 6 (60.0%)  3 (30.0%)  1 (10.0%)  - | 9 (90.0%)  1 (10.0%)  -  - | 9 (90.0%)  -  -  1 (10.0%) | 24 (80.0%)  4 (13.3%)  1 (3.3%)  1 (3.3%) |
| **Diagnosis of asthma, n (%)**  No  Yes | 7 (70.0%)  3 (30.0%) | 10 (100%)  - | 8 (80.0%)  2 (20.0%) | 25 (83.3%)  5 (16.7%) |
| **Recruitment site, n (%)**  Chicago, IL  Baltimore, MD  St. Louis, MO  New Orleans, LA  Pittsburgh, PA | 5 (50.0%)  5 (50.0%)  -  -  - | 4 (40.0%)  -  3 (30.0%)  2 (20.0%)  1 (10.0%) | 7 (70.0%)  1 (10.0%)  2 (20.0%)  -  - | 16 (53.3%)  6 (20.0%)  5 (16.7%)  2 (6.7%)  1 (3.33%) |

^1^All participants identified gender was the same as their sex.

^2^Some participants selected more than one response.

| **Supplementary Table 2: Symptom and impact level saturation grid for child participants across 3 rounds of interviews (n=15)** | | | | | | | | | | | | | | | | |
| --- | --- | --- | --- | --- | --- | --- | --- | --- | --- | --- | --- | --- | --- | --- | --- | --- |
| **Participant** | **Round 1** | | | | | **Round 2** | | | | | **Round 3** | | | | | **Total (n=15)** |
|  | **01-40-M-11-WC-P** | **03-40-M-8-WC-P** | **06-20-M-8-WC-P** | **09-20-F-10-NWC-P** | **10-40-F-9-WC-P** | **13-10-M-11-NWC-P** | **14-10-M-8-NWC-P** | **16-40-F-8-NWC-P** | **19-40-M-9-WC-P** | **18-50-M-9-NWC-P** | **23-40-M-9-NWC-P** | **27-40-F-11-NWC-P** | **30-40-F-11-NWC-P** | **33-40-M-10-WC-P** | **34-50-F-9-NWC-P** |  |
| **Signs/Symptoms** | | | | | | | | | | | | | | | | |
| Chest pain | **S** | - | - | - | - | - | - | - | - | - | - | - | - | - | - | **1** |
| Chest tightness | P | **S** | P | S | S | P | P | S | P | S | P | P | P | P | P | **15** |
| Cough | P | P | **S** | P | S | S | P | P | P | S | P | S | S | P | P | **15** |
| Difficulty breathing | **S** | P | P | S | S | S | S | S | P | S | S | S | S | S | S | **15** |
| Pain | - | - | - | - | - | **S** | - | - | - | - | - | - | - | - | - | **1** |
| Headache | - | - | **S** | - | - | - | - | - | - | - | - | - | - | - | - | **1** |
| Shortness of breath | **S** | P | P | P | P | P | P | P | - | P | S | S | P | P | P | **14** |
| Stomach tightness | - | - | - | - | - | **S** | - | - | - | - | - | - | - | - | - | **1** |
| Stuffed nose | - | - | - | - | - | **S** | S | - | - | - | - | - | - | - | - | **2** |
| Weak body | - | - | - | - | - | - | - | - | - | - | - | - | - | **S** | - | **1** |
| Wheeze | **S** | P | P | P | P | P | P | P | P | S | S | P | S | - | P | **14** |
| **Impact domain** | | | | | | | | | | | | | | | | |
| Emotional | - | - | **S** | S | - | S | - | - | S | S | - | - | - | - | - | **5** |
| Physical | **S** | S | S | S | S | S | P | S | S | S | S | S | S | S | S | **15** |
| School | - | - | - | - | - | **S** | S | - | - | - | S | - | - | - | - | **3** |
| Difficulty falling asleep | P | P | P | P | P | P | **S** | P | P | S | P | P | P | P | - | **14** |
| Nighttime awakenings | P | P | - | P | P | P | P | P | P | P | P | **S** | P | P | P | **14** |
| Social | P | **S** | S | - | - | S | - | S | - | S | S | - | - | - | - | **7** |
| **Total per participant** | **10** | **9** | **10** | **9** | **8** | **14** | **10** | **9** | **8** | **10** | **10** | **8** | **8** | **8** | **7** | **138** |

S = symptom/impact concept reported spontaneously; P = symptom/impact concept reported when probed.

The table cells where **S** is bolded and underlined indicate the first spontaneous report of each concept.

Concept saturation was deemed to have been achieved if no new significant symptom or impact concepts were spontaneously reported in the final round of interviews.

Note that, when referring to a participant, “M” or “F” indicates the sex of the participant, “WC” indicates well-controlled asthma, “NWC” indicates not well-controlled asthma, and “P” indicates that the participant is a pediatric participant.

| Supplementary Table 3: Symptom and impact level saturation grid for caregiver participants across 3 rounds of interviews (n=30) | | | | | | | | | | | | | | | | | | | | | | | | | | | | | | | |
| --- | --- | --- | --- | --- | --- | --- | --- | --- | --- | --- | --- | --- | --- | --- | --- | --- | --- | --- | --- | --- | --- | --- | --- | --- | --- | --- | --- | --- | --- | --- | --- |
|  | **Round 1** | | | | | | | | | | **Round 2** | | | | | | | | | | **Round 3** | | | | | | | | | | **Total (n=30)** |
| **Participant** | **03-40-F-39-WC-CG** | **02-40-M-42-WC-CG** | **04-20-M-47-NWC-CG** | **06-20-M-30-WC-CG** | **01-40-F-51-WC-CG** | **05-20-F-24-NWC-CG** | **07-20-F-34-NWC-CG** | **08-40-F-38-NWC-CG** | **10-40-F-38-WC-CG** | **09-20-F-35-NWC-CG** | **13-10-F-36-NWC-CG** | **14-10-F-45-NWC-CG** | **15-40-F-40-WC-CG** | **16-40-F-36-NWC-CG** | **19-40-F-31-WC-CG** | **11-30-F-34-WC-CG** | **20-50-F-24-NWC-CG** | **12-40-F-29-WC-CG** | **18-50-F-34-NWC-CG** | **17-50-F-30-WC-CG** | **21-40-M-41-NWC-CG** | **23-40-F-36-NWC-CG** | **29-40-F-54-NWC-CG** | **34-50-F-31-NWC-CG** | **28-40-M-42-NWC-CG** | **26-50-F-27-NWC-CG** | **32-40-M-52-NWC-CG** | **24-40-F-42-NWC-CG** | **22-40-F-25-WC-CG** | **25-20-F-70-WC-CG** |  |
| **Signs/Symptoms** | | | | | | | | | | | | | | | | | | | | | | | | | | | | | | | |
| Cough | P | **S** | S | S | S | S | S | S | S | S | S | S | P | P | S | S | S | P | S | S | P | S | P | P | S | S | P | P | S | S | **30** |
| Difficulty breathing | **S** | S | S | P | S | S | S | S | S | S | S | P | S | S | P | P | P | S | S | S | P | S | P | S | P | S | S | S | P | S | **30** |
| Shortness of breath | **S** | S | P | S | P | S | P | S | S | P | S | P | S | P | S | - | P | - | S | S | P | S | S | S | S | P | S | S | S | P | **28** |
| Wheeze | **S** | P | S | S | P | S | S | S | S | P | S | S | P | P | S | P | S | S | S | P | S | S | S | P | S | S | P | S | S | P | **30** |
| Chest tightness | - | - | - | - | - | - | - | - | - | - | - | - | **S** | S | - | - | - | - | - | - | - | S | - | S | - | - | - | - | - | - | **4** |
| Dizziness | - | **S** | - | - | - | - | - | - | - | - | - | - | - | - | - | - | - | - | - | - | - | - | - | - | - | - | - | - | - | - | **1** |
| Flushed face | - | - | - | - | - | **S** | - | - | - | - | - | - | S | - | - | - | - | - | - | - | - | - | - | - | - | - | - | - | - | - | **2** |
| Runny nose | - | - | - | - | - | **S** | - | - | - | - | - | - | - | - | - | - | - | - | - | - | - | - | - | - | - | - | - | - | - | - | **1** |
| Congestion | - | - | - | - | - | - | - | - | - | - | - | **S** | - | - | - | - | - | - | - | - | - | S | S | - | - | - | - | - | - | - | **3** |
| Heart murmur | - | - | - | - | - | - | - | - | - | - | - | - | - | - | - | - | - | **S** | - | - | - | - | - | - | - | - | - | - | - | - | **1** |
| Heart racing | - | - | - | - | - | - | - | - | - | - | - | - | - | - | - | - | - | - | **S** | - | - | - | - | - | - | - | - | - | - | - | **1** |
| Low oxygen | - | - | - | - | - | - | - | - | - | - | **S** | - | - | - | - | - | - | - | - | - | - | - | - | - | - | - | - | - | - | - | **1** |
| Muscles hurt | - | - | - | - | - | - | - | - | - | - | - | - | - | - | - | **S** | - | - | - | - | - | - | - | - | - | - | - | - | - | - | **1** |
| Pale face | - | - | - | - | - | - | - | - | - | - | - | - | **S** | - | - | - | - | - | - | - | - | - | - | - | - | - | - | - | - | - | **1** |
| Shakiness | - | - | - | - | - | - | - | - | - | - | - | - | - | - | - | - | - | - | **S** | - | - | - | - | - | - | - | - | - | - | - | **1** |
| Sweating | - | - | - | - | - | - | - | - | - | - | - | - | - | - | **S** | - | - | - | - | - | - | - | - | - | - | - | - | - | - | - | **1** |
| Tiredness | - | - | - | - | - | - | - | - | - | - | - | - | **S** | - | - | - | - | S | - | S | - | - | - | - | - | - | - | - | - | - | **3** |
| Dry mouth | - | - | - | - | - | - | - | - | - | - | - | - | - | - | - | - | - | - | - | - | - | - | - | - | - | - | **S** | - | - | - | **1** |
| Lazy eye | - | - | - | - | - | - | - | - | - | - | - | - | - | - | - | - | - | - | - | - | - | - | - | - | - | - | - | - | - | **S** | **1** |
| Raspy voice | - | - | - | - | - | - | - | - | - | - | - | - | - | - | - | - | - | - | - | - | - | - | - | - | - | - | **S** | - | - | - | **1** |
| Throat/ mouth pain | - | - | - | - | - | - | - | - | - | - | - | - | - | - | - | - | - | - | - | - | - | - | - | - | - | - | - | **S** | - | - | **1** |
| **Impact Domain** | | | | | | | | | | | | | | | | | | | | | | | | | | | | | | | |
| Emotional | - | **S** | S | - | - | S | - | - | - | S | - | - | S | - | S | S | S | S | - | - | - | - | S | - | S | S | - | - | - | - | **12** |
| Physical | P | **S** | S | S | S | S | S | S | S | S | S | S | S | S | S | P | S | S | S | S | S | S | S | S | S | S | S | S | S | P | **30** |
| Nighttime awakenings | - | P | **S** | - | P | S | S | P | S | P | S | P | P | P | P | P | S | P | S | S | P | P | P | P | P | P | S | - | P | P | **27** |
| Difficulty falling asleep | - | P | P | P | - | - | P | P | P | P | P | P | P | P | P | **S** | P | P | P | P | P | P | P | P | P | P | P | P | P | P | **27** |
| Social | - | **S** | - | S | S | - | S | - | - | - | P | - | S | - | - | - | - | S | S | - | S | S | - | - | - | - | - | - | - | P | **11** |
| School | - | - | - | - | - | - | - | - | - | - | - | - | - | - | - | - | - | - | - | - | - | **S** | - | - | - | - | - | - | - | - | **1** |
| **Total per participant** | 5 | 10 | 8 | 7 | 7 | 9 | 8 | 7 | 7 | 8 | 9 | 8 | 13 | 8 | 9 | 8 | 8 | 9 | 10 | 8 | 8 | 11 | 9 | 8 | 8 | 8 | 9 | 7 | 7 | 9 | **250** |

S = symptom/impact concept reported spontaneously; P = symptom/impact concept reported when probed.

The table cells where **S** is bolded and underlined indicate the first spontaneous report of each concept.

Concept saturation was deemed to have been achieved if no new significant symptom or impact concepts were spontaneously reported in the final round of interviews.

Note that, when referring to a participant, “M” or “F” indicates the sex of the participant, “WC” indicates well-controlled asthma, “NWC” indicates not well-controlled asthma, and “CG” indicates that the participant is a caregiver.

Supplementary Figure 1: Participant understanding of the *PAD-C* Morning Diary items by percentage of participants

^1^ Edits made between rounds which were tested with all 15 participants, although slightly different iterations may have been tested in each round

^2^ Added after Round 1 interviews and tested with 10 participants only

Note that instructions/items with no asterisks were not edited between rounds and were tested with all 15 participants.

Supplementary Figure 2: Participant understanding of the *PAD-C* Bedtime Diary items by percentage of participants

^1^ Edits made between rounds which were tested with all 15 participants, although slightly different iterations may have been tested in each round

^2^ Added after Round 1 interviews and tested with 10 participants only

Note that instructions/items with no asterisks were not edited between rounds and were tested with all 15 participants.

Supplementary Figure 3: Relevance of the *PAD-C* Morning Diary items by percentage of participants

^1^ Edits made between rounds which were tested with all 15 participants, although slightly different iterations may have been tested in each round

^2^ Added after Round 1 interviews and tested with 10 participants only

Note that items with no asterisks were not edited between rounds and were tested with all 15 participants.

Supplementary Figure 4: Relevance of the *PAD-C* Bedtime Diary items by percentage of participants

^1^ Edits made between rounds which were tested with all 15 participants, although slightly different iterations may have been tested in each round

^2^ Added after Round 1 interviews and tested with 10 participants only

Note that items with no asterisks were not edited between rounds and were tested with all 15 participants

Supplementary Figure 5: Participant understanding of the *PAD-O* Morning Diary by percentage of participants

^1^ Edits made between rounds which were tested with all 30 participants, although slightly different iterations may have been tested in each round

^2^ Added after Round 1 interviews and tested with 20 participants only

^3^ Added after Round 2 interviews and tested with 10 participants only

Note that instructions/items with no asterisks were not edited between rounds and were tested with all 30 participants.

Supplementary Figure 6: Participant understanding of the *PAD-O* Evening Diary by percentage of participants

^1^ Edits made between rounds which were tested with all 30 participants, although slightly different iterations may have been tested in each round

^2^ Added after Round 1 interviews and tested with 20 participants only

^3^ Added after Round 2 interviews and tested with 10 participants only

Note that instructions/items with no asterisks were not edited between rounds and were tested with all 30 participants.

Supplementary Figure 7: Relevance of the *PAD-O* Morning Diary items by percentage of participants

^1^ Edits made between rounds which were tested with all 30 participants, although slightly different iterations may have been tested between rounds

^2^ Added after Round 1 interviews and tested with 20 participants only

Note that items with no asterisks were not edited between rounds and were tested with all 30 participants.

Supplementary Figure 8: Relevance of the *PAD-O* Evening Diary items by percentage of participants

^1^ Edits made between rounds which were tested with all 30 participants, although slightly different iterations may have been tested between rounds

^2^ Added after Round 1 interviews and tested with 20 participants only

Note that items with no asterisks were not edited between rounds and were tested with all 30 participants.
